# Serum Uric Acid Associates with Systemic Complement C3 Activation in Severe ANCA-Associated Renal Vasculitides

**DOI:** 10.3390/ijms25020713

**Published:** 2024-01-05

**Authors:** Eva Baier, Ingmar Alexander Kluge, Samy Hakroush, Peter Korsten, Björn Tampe

**Affiliations:** 1 Department of Nephrology and Rheumatology, University Medical Center Göttingen, 37075 Göttingen, Germany; eva.baier@med.uni-goettingen.de; 2 Institute of Pathology, University Medical Center Göttingen, 37075 Göttingen, Germany; ingmar.kluge@med.uni-goettingen.de (I.A.K.); samy.hakroush@med.uni-goettingen.de (S.H.); 3 SYNLAB Pathology Hannover, SYNLAB Holding Germany, 86156 Augsburg, Germany; 4 Institute of Pathology, Klinikum Bremen-Mitte, School of Medicine of the University of Göttingen, 28205 Bremen, Germany; 5 Department of Rheumatology and Clinical Immunology, St. Josef-Stift Sendenhorst, 48324 Sendenhorst, Germany; peter.korsten@med.uni-goettingen.de

**Keywords:** serum uric acid, innate immunity, complement activation, complement C3, ANCA-associated renal vasculitis, anti-neutrophil cytoplasmic antibody

## Abstract

Involvement of the complement system is key to the pathogenesis of antineutrophil cytoplasmic antibody (ANCA)-associated renal vasculitis, but immunometabolic implications, especially on serum uric acid (UA) levels, still need to be elucidated. A total of 34 patients with biopsy-proven ANCA-associated renal vasculitis between 2015 and 2020 were retrospectively enrolled. Serum UA levels were correlated with clinical and histopathological characteristics, separated for critically ill (CI, *n* = 19), myeloperoxidase (MPO)-ANCA (*n* = 21) and proteinase 3 (PR3)-ANCA (*n* = 13) subgroups. We here identified inverse correlations of serum UA levels and complement C3 levels in the total cohort (*p* = 0.005) and the CI subgroup (*p* < 0.001). Intrarenal complement C4d deposition in venules correlated with serum UA levels in the total cohort (*p* = 0.007) and in the CI subgroup (*p* = 0.016). Significant associations of serum UA levels and tubulitis in areas of scarred cortex (*t-IFTA*) were identified in the total cohort (*p* = 0.008), and both subgroups of CI (*p* = 0.034) and MPO-ANCA (*p* = 0.029). In PR3-ANCA, interstitial fibrosis (*ci*) was observed as the strongest association with serum UA levels (*p* = 0.022). Our observations broaden our current understanding of contributory metabolic factors that influence the initial disease course in ANCA-associated renal vasculitis.

## 1. Introduction

Being classified as a small vessel vasculitis (SVV), antineutrophil cytoplasmic antibody (ANCA)-associated vasculitis (AAV) is a severe autoimmune disease that may present as granulomatosis with polyangiitis (GPA), microscopic polyangiitis (MPA), or eosinophilic granulomatosis with polyangiitis (EGPA), according to the 2012 revised nomenclature of vasculitides of the Chapel Hill Consensus Conference [1]. Renal involvement is a major predictor of overall mortality in AAV patients, and the term ANCA-associated renal vasculitis refers to a kidney-focused view of mainly GPA and MPA patients with biopsy-proven kidney affection [2]. While pauci-immune crescentic and necrotizing glomerulonephritis is commonly seen in renal biopsies, clinical manifestation of ANCA-associated renal vasculitis ranges from rapid deterioration of kidney function requiring kidney replacement therapy (KRT) to chronic kidney disease (CKD), progressing to end-stage kidney disease (ESKD) [2,3]. Different epitopes on neutrophils, namely myeloperoxidase (MPO) and proteinase 3 (PR3), are mechanistically implied in the pathophysiology of ANCA-associated renal vasculitis, and ANCA binding to these epitopes leads to an activation of neutrophils engaging in so-called NETosis, the formation of neutrophilic extracellular traps (NETs) [4].

In AAV with renal involvement, the state of elevated serum UA levels, hyperuricemia (HU), was demonstrated to be an independent risk factor of progression to end-stage kidney disease (ESKD) [5,6]. Serum uric acid (UA) is the final metabolite of purine nucleotide degradation and due to a lack of the enzyme uricase, humans and higher primates feature three times higher levels of serum UA in comparison to other species, which are capable of producing the more water-soluble allantoin [7]. In humans, serum UA is implied in regulating oxidative stress, on the one hand serving as a powerful reactive radical scavenger that protects cells from oxidative damage and accounts for approximately half of the antioxidant capacity of human plasma, but on the other hand, when oxidized to its radical, serum UA itself can function as a pro-oxidant activating inflammation, amongst others, via the Iκ-B/NFκB signaling cascade [8,9,10,11].

Approximately two thirds of UA metabolites undergo renal elimination, and decreased renal function was shown to correlate with HU in patients with MPA and GPA [6]. Even though serum UA is concededly referred to as an inflammation-modulating component of the vascular microenvironment, clinicopathological correlations of serum UA with respect to laboratory parameters reflecting systemic inflammation and vasculitis manifestations within kidney biopsies in ANCA-associated renal vasculitis lack further lucidity. We therefore systematically assessed clinicopathological associations of serum UA levels with clinical, laboratory, and histopathological parameters in ANCA-associated renal vasculitis.

## 2. Results

### 2.1. Baseline Characteristics

**Figure 1:**
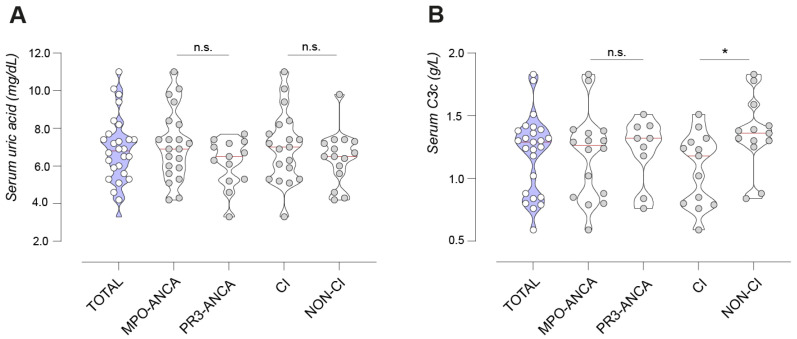
Serum UA and complement C3 levels in the total cohort and subgroups of patients with ANCA-associated renal vasculitides. (**A**,**B**) Violin plots with medians and data points for serum UA levels (**A**) and serum complement C3 levels (**B**) in ANCA-associated renal vasculitis; in the total cohort and subgroups MPO-ANCA, PR3-ANCA, CI and non-CI. Subgroup comparison was performed between MPO-ANCA and PR3-ANCA, with CI and non-CI. Abbreviations: ANCA, anti-neutrophil cytoplasmic antibody; C3, complement factor 3; CI, critically ill; MPO, myeloperoxidase; PR3, proteinase 3; UA, uric acid. * *p* < 0.05, n.s. not significant.

The baseline characteristics of the total cohort and three subgroups are itemized in Table 1. Enrolled patients featured a median age of 69 years, with 41.4% (14/34) being female; they had well-controlled blood pressure levels at the time of renal biopsy and were slightly overweight, with a BMI of 26 ± 5 kg/m^2^. Among 34 patients, 9/34 (26.5%) presented with HU, and aHT was very common at 85.3% (29/34), while dDM and cAA seldom occurred (Table 1). Except for the occurrence of aHT (*p* = 0.0375, Table 1), BMI (*p* = 0.0417, Table 1), and usage of diuretics (*p* = 0.0416, Table 1), we found no significant subgroup differences regarding clinical factors contributing to metabolic status, e.g., age, sex, treatment with XOi, statins, or occurrence of cAA, dDM, and HU (Table 1). Serum UA levels averaged out at 6.8 ± 1.7 mg/dL with no significant subgroup differences (*p* = 0.9999, Table 1 and Figure 1A). Median creatinine levels at admission accounted for 2.9 mg/dL in the total cohort, while in CI, the highest median with 4.2 mg/dL was observed (Table 1). Serum C3 levels with a total mean of 1.2 ± 0.3 g/L only featured significant subgroup differences when CI was compared to non-CI (*p* = 0.018, Figure 1B and Table 1). Concerning histopathological items, normal glomeruli showed a significantly higher fraction in PR3-ANCA (*p* = 0.0094, Table 1), while the distribution pattern of lesions analogous to the Banff score and intrarenal complement deposition featured no significant subgroup differences (Table 1).

### 2.2. Serum UA Levels Are Inversely Associated with Serum C3 Complement Levels, Especially in Critically Ill Patients with ANCA-Associated Renal Vasculitis

**Figure 2:**
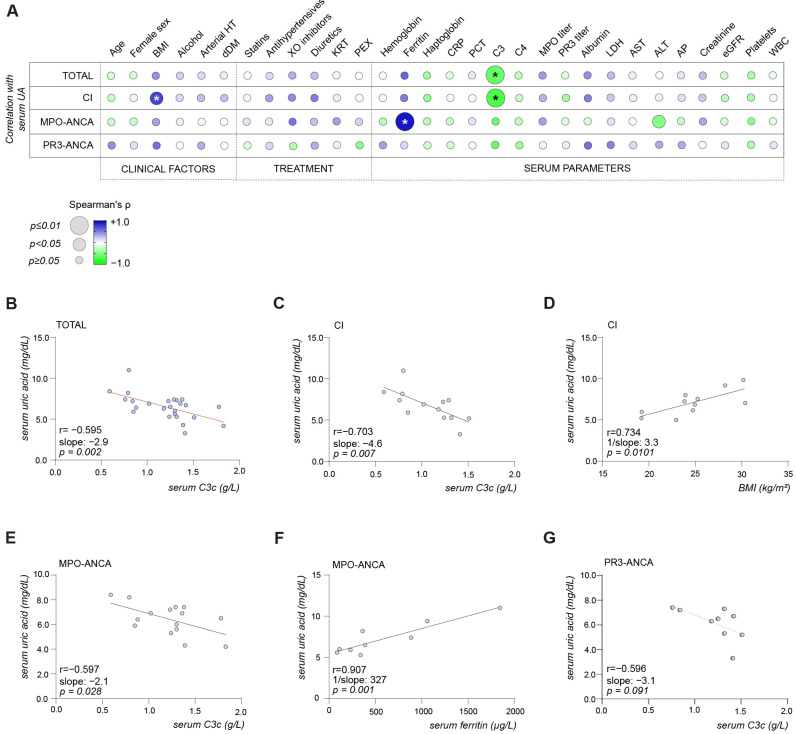
Serum UA levels are associated with decreased complement C3 levels, especially in critically ill patients with ANCA-associated renal vasculitides. (**A**) Correlation matrix shows mean values of Spearman’s/Pearson’s r; significant *p* values in the Pearson’s/Sperman’s correlation are marked with an asterisk, and circle size represents significance level in the stepwise multivariable linear regression. (**B**–**G**) Simple linear regressions between serum UA levels and serum parameters in the total cohort (**B**), CI (**C**,**D**), MPO-ANCA (**E**,**F**), PR3-ANCA (**G**). Slope is indicated by a red line. Abbreviations: ALT, alanine aminotransferase; ANCA, anti-neutrophil cytoplasmic antibody; AP, alkaline phosphatase; arterial HT, arterial hypertension; AST, aspartate aminotransferase; BMI, body mass index; C3, complement factor 3; C4, complement factor 4; CI, critically ill; CRP, C-reactive protein; dDM, decompensated diabetes mellitus; eGFR, estimated glomerular filtration rate; KRT, kidney replacement therapy; LDH, lactate dehydrogenase; MPO, myeloperoxidase; PCT, procalcitonin; PEX, plasma exchange; PR3, proteinase 3; UA, uric acid; WBC, white blood cells; XO inhibitors, xanthine oxidase inhibitors.

First, we analyzed factors that commonly influence UA metabolism in correlation with serum UA levels in ANCA-associated renal vasculitis. Therefore, we included clinical factors (BMI, age, female sex, cAA, aHT, dDM, treatment with diuretics/antihypertensives, XOi, statins) and laboratory parameters reflecting systemic inflammation (ferritin, haptoglobin, CRP, PCT, WBC, complement C3/C4 levels, PR3- and MPO-titers), as well as parameters indicative of kidney injury. In the total cohort, the variables BMI, serum albumin levels, and complement C3 levels qualified for stepwise multivariable linear regression analysis using serum UA as outcome variable. Here, an inverse correlation of serum UA levels and complement C3 levels was identified as the strongest association (r = −0.595; β = −0.462, *p* = 0.005, Figure 2A and Table 2), which was further confirmed by simple linear regression (slope = −2.9, *p* = 0.002, Figure 2B). In CI, the independent variables treatment with diuretics, serum albumin levels, BMI, and complement C3 levels were included in the stepwise multivariable regression analysis (Table 2). The strongest association was observed between serum UA and C3 levels (r = −0.703; β = −0.700, *p* < 0.001, Figure 2A and Table 2). Simple linear regression was also significant (slope = −4.6, *p* = 0.007, Figure 2C). When studied in a model including the variables of serum levels of ferritin, platelet counts, ALT, treatment with XOi, BMI, and complement C3 levels in MPO-ANCA (Table 2), ferritin levels (r = 0.907; β = 0.598, *p* = 0.004, Figure 2A and Table 2; 1/slope: 327, *p* = 0.001, Figure 2F) featured the strongest association with the outcome variable of serum UA levels, while serum complement C3 levels missed statistical significance (r = −0.597; β = −0.244, *p* = 0.203, Figure 2A and Table 2).

### 2.3. Histopathological Associations with Serum UA Levels in ANCA-Associated Renal Vasculitis

**Figure 3:**
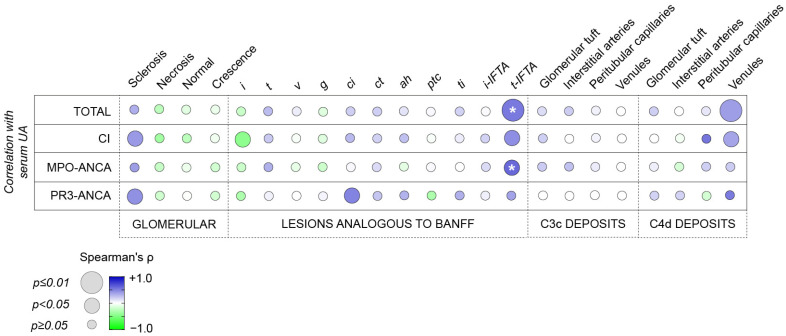
Tubulitis in areas of scarred cortex correlates with serum UA levels in ANCA-associated renal vasculitides. Correlation matrix shows mean values of Spearman’s/Pearson’s r; significant *p* values in Pearson’s/Spearman’s correlation are marked with an asterisk, and circle size represents significance level in the stepwise multivariable linear regression. Abbreviations: *ah*, arteriolar hyalinosis; ANCA, anti-neutrophil cytoplasmic antibody; CI, critically ill; *ci*, interstitial fibrosis; *ct*, tubular atrophy; *g*, glomerulitis; *i*, interstitial inflammation; *IFTA*, interstitial fibrosis/tubular atrophy; *i-IFTA*, inflammation in areas of IFTA; MPO, myeloperoxidase; PR3, proteinase 3; *ptc*, peritubular capillaritis; *t*, tubulitis; *ti*, total inflammation; *t-IFTA*, tubulitis in areas of IFTA; UA, uric acid; *v*, intimal arteritis.

Finally, we correlated serum UA levels and intrarenal histopathological lesions including glomerular aberrancies in terms of glomerulosclerosis, necrosis, crescentic formations, lesions analogous to the Banff score, and intrarenal complement deposition of C3c and C4d. Significant associations of serum UA levels and *t-IFTA* were identified in the total cohort (r = 0.524; β = 0.494, *p* = 0.008, Figure 3 and Table 3), in CI (r = 0.443; β = 0.488, *p* = 0.034, Figure 3 and Table 3), and in MPO-ANCA (r = 0.590; β = 0.488, *p* = 0.029, Figure 3 and Table 3). Moreover, we identified serum UA levels to feature the strongest correlation with C4d complement deposition of venules in the total cohort (r = 0.380; β = 0.500, *p* = 0.007, Figure 3 and Table 3) and in CI (r = 0.356; β = 0.513, *p* = 0.016, Figure 3 and Table 3). In PR3-ANCA, interstitial fibrosis (*ci*) was observed as the strongest association with serum UA levels (r = 0.484; β = 0.626, *p* = 0.022, Figure 3 and Table 3).

## 3. Discussion

In this study, we aimed to identify clinicopathological correlations of serum UA levels in hospitalized patients with ANCA-associated renal vasculitis under special attention given their critical illness and seropositivity for MPO-ANCA and PR3-ANCA. First, we identified serum UA levels to independently correlate with decreased complement serum C3 levels in the total cohort and the CI subgroup, thus providing evidence that serum UA is implied in the activation of the complement system in ANCA-associated renal vasculitis, especially in critically ill patients. Based on increasing evidence, systemic complement perturbation reflected by decreased serum C3 levels is prognostically relevant for patients with ANCA-associated renal vasculitis, even though hypocomplementemia per se seldom occurs [12,13,14,15]. In a study by Brilland et al., decreased complement C3 levels below the median level of 1.21 g/L were observed in patients that experienced either KRT within the first 90 days from diagnosis, progression to ESKD, or death during a follow-up period of 60 months [14]. These observations were independently confirmed with lower complement C3 levels being the strongest negative outcome predictor for death or ESKD at 1 year, as observed by Scurt et al. [16]. In line with our recently published study, patients with low C3 levels featured worse renal function and higher proteinuria levels [13,14]. Moreover, Lionaki et al. implicated decreased serum C3 levels to correlate with resistance to immunosuppressive treatment [15].

There are several interconnecting points between UA metabolism and innate immune responses. Serum UA functions as a powerful antioxidant, thus moderating intravascular inflammation; however, when oxidized to its radical, serum UA itself can act in a pro-inflammatory manner, e.g., by activation of the Iκ-B/NF-κB signaling pathway [8,9,11]. Ma et al. described an immunomodulatory effect of soluble UA by preventing β2 integrin-mediated neutrophilic recruitment [7]. Contrasting these findings, crystallized UA in terms of monosodium urate (MSU) crystals are considered immunostimulatory, while in the field of tophaceous gout, it is known that deposition of MSU crystals requires a second factor to evoke an inflammatory condition, which is involvement of the complement system [17]. The pentraxin CRP is commonly involved in opsonization, leading to complement activation and phagocytosis [18]. In gout, CRP opsonizes MSU crystals, thus activating the classical and lectin pathway of the complement system by recruitment of C1 [19,20]. The anaphylatoxin C5a was shown to potentiate MSU crystal-induced production of IL-1β [17]. NETs and MSU crystals compose gout tophi, and neutrophilic ingestion of MSU crystals leads to NETosis, wherein a sudden resolution of gout attack is mapped to the formation of so-called aggregated NETs, which are potent in trapping and turning down inflammatory factors [21]. Moreover, increased levels of serum UA were demonstrated to promote MPO release from neutrophils, and in turn, oxidation of serum UA [10]. On a mechanistic level, UA is implied in adaptive immune responses [22]. In the presence of the aforementioned NF-κB signaling, MSU crystals promote cytokine production via antigen-presenting cells (APC), specifically dendritic cells (DC), driving polarization of IL-17A-producing CD4-positive T helper cells (T_H_17), which in turn are essential for crescentic glomerulonephritis because they drive neutrophil-mediated inflammation [22,23,24,25]. In vitro and in vivo approaches showed a MSU crystal-associated T_H_17 induction affecting IL-1β signaling [22]. However, a direct linkage between serum UA and T_H_17 activity remains elusive.

In the field of critical care research, serum UA was repeatedly related to prediction of mortality in critically ill patients. During the COVID-19 pandemic, the lowest serum UA levels independently predicted in-hospital death in a Chinese cohort of 540 patients with severe and critical COVID-19 [26]. In line with other studies, the authors observed low serum UA levels and higher inflammatory levels in patients that died, and concluded that low serum UA levels reflect elevated inflammatory status [26,27]. Contrasting to this, HU on arrival to the intensive care unit (ICU) was associated with poor prognosis in patients with (mostly bacteria-induced) sepsis [28]. In a prospective cohort study with 144 enrolled patients, elevated serum UA levels were shown to be associated with a higher risk of acute kidney injury (AKI), and an increased severity of illness assessed by established ICU scores [28]. Mechanistically, the authors attributed serum UA causing AKI to the secondary vasoconstrictive effect of serum UA, which results from an activation of the renin–angiotensin system, vasoactive mediators, elevated oxidative stress, and decreased nitric oxide levels [28]. In a rat model, UA inhibits nitric oxide release from endothelial cells [29].

In our correlative analyses, we included potential confounding factors such as patients’ age, sex, BMI, current alcohol consumption, treatment with diuretics, antihypertensives, statins and XO inhibitors. Contrasting other studies in which correlative analyses between serum UA levels and laboratory parameters were performed, we included a broad spectrum of laboratory parameters that reflect ongoing systemic inflammation, involvement in iron metabolism, and other influencing aspects. Behind the background of serum UA being implied in iron metabolism, our second main finding of an independent correlation between serum UA and ferritin levels in MPO-ANCA appears intelligible, while a positive correlation between serum UA and ferritin levels was repeatedly implied [30,31]. For coping with oxidative stress, nonenzymatic defense mechanisms encompassing metal-binding proteins (such as haptoglobin and albumin) and free radical scavengers like serum UA are required to maintain redox homeostasis, which is disturbed in diseases affecting iron metabolism, such as thalassemia [30]. The reason that this correlation occurred in the MPO-positive subgroup, while the association of serum C3 levels and serum UA missed statistical significance, remains speculative. In healthy individuals, it was shown that serum C3 levels and ferritin levels feature a positive correlation [32]. In dialysis patients, application of IV iron resulted in elevated levels of MPO, and relative increase in MPO levels correlated with markers of complement activation assessed by sC5b-9 [33].

We also showed herein that tubulitis in areas of scarred cortex is associated with serum UA levels, especially in CI and MPO-ANCA, while a correlation between interstitial fibrosis and serum UA levels was observed in PR3-ANCA. Under physiological conditions, UA transporters located in tubular epithelial cells reabsorb UA, which is freely filtered in the glomeruli [28]. Upon stimulation with UA, tubular epithelial cells were shown to enhance the expression of inflammatory mediators of the NF-κB pathway [34]. While up-regulation of the chemokine monocyte-chemotactic protein 1 (MCP-1) was reported to be enhanced in HU mice kidneys that further featured increased T cell and macrophage infiltration largely located in tubular interstitial areas, MCP-1 in particular has been implied as an urinary biomarker of active or relapsing renal vasculitis in the past [34,35,36,37,38,39].

As a fourth main aspect of this study, complement C4d deposition of venules correlated with serum UA levels in the total cohort and the CI subgroup. We have shown that complement C3 deposition in the glomerular tuft is associated with impaired kidney function in ANCA-associated renal vasculitis [12,40]. Independent of consumption of systemic complement components, CRP levels correlated with active renal lesions and intrarenal C4 deposition in interstitial arteries in MPO-ANCA renal vasculitis [41]. We saw an implication for CRP engaging in intrarenal complement synthesis. Additionally, in this study, we found that UA may be involved in intrarenal complement synthesis. We are aware that the patient numbers were small, and follow-up studies will be needed to corroborate these findings.

## 4. Materials and Methods

### 4.1. Study Population and Data Assessment

A total of 34 patients with available serum UA levels and biopsy-proven ANCA-associated renal vasculitis between 2015 and 2020 at the University Medical Center Göttingen (UMG), Göttingen, Germany, were retrospectively enrolled. The patient cohort has recently been described in part [12,40,42]. The institutional review board of the UMG reviewed and approved this study, which involves human participants (protocol no. 4/8/19). As part of the regular medical care in the UMG contract, all enrolled patients provided their written informed consent for the scientific utilization of routine data collection. Based on the medical records, assessment of clinical data included date of admission, discharge and transfer within the hospital, age, sex, and blood pressure levels at renal biopsy. To cover a broad spectrum of putative factors influencing serum UA levels, comorbidities and other variables reflecting the metabolic status were assessed (body mass index [BMI], arterial hypertension [aHT], decompensated diabetes mellitus [dDM], HU, current alcohol abuse [cAA]), and treatment measures affecting serum UA levels, such as application of diuretics, xanthine oxidase inhibitors (XOi, also called uricostatics: allopurinol, and febuxostat), statins, and antihypertensives (angiotensin-converting enzyme inhibitors, angiotensin receptor blockers, beta blockers and calcium antagonists); dDM was defined as pre-diagnosed diabetes mellitus in addition to currently elevated HbA1_c_ levels above 7%. HU was defined as serum UA levels of greater than 6.7 mg/dL for female patients and greater than 7.4 mg/dL for male patients. The Birmingham Vasculitis Activity Score (BVAS) and the Simplified Acute Physiology Score II (SAPS II) were calculated as previously described [43,44]. The state of critical illness (CI) was defined as recently described [42,45]. Laboratory data assessment encompassed whole blood cell counts (WBC) including hemoglobin levels; platelet and leukocyte counts; and serum levels of inflammatory markers, such as C-reactive protein (CRP), ferritin, haptoglobin, procalcitonin (PCT) and albumin; serum creatinine and blood urea nitrogen (BUN) levels at admission; iron; lactate dehydrogenase (LDH); aspartate amino transferase (AST); alanine amino transferase (ALT); and alkaline phosphatase (AP). For eGFR calculation, the CKD-EPI (chronic kidney disease epidemiology collaboration) equation was used [46]. Using turbidimetric measurements on the ARCHITECT-C module, serum concentrations of human complement components C3 (9D9621, Abbott, Chicago, IL, USA) and C4 (9D9721, Abbott, Chicago, IL, USA) were assessed. Immunoassays (ImmunoCAP 250, Thermo Fisher Scientific, Waltham, MA, USA) were used to assess serum levels of MPO-ANCA and PR3-ANCA. According to the manufacturer’s protocol, ANCA immunofluorescence was performed (EUROIMMUN AG, Lübeck, Germany). Three subgroups were formed: CI (*n* = 19), MPO-ANCA (*n* = 21), and PR3-ANCA (*n* = 13). For improved readability, the short form (“in CI/MPO-ANCA/PR3-ANCA”) will be used for referring to respective subgroups.

### 4.2. UA Measurements

Serum concentrations of UA (3P39-21, Abbott, Chicago, IL, USA) were determined by turbidimetric measurements on the ARCHITECT-C module. Reference range serum concentrations for UA are defined between 2.6 to 6.0 mg/L for adult women, and 3.5 to 7.2 mg/L for adult men.

### 4.3. ANCA Autoantibody and Complement Measurements

MPO-ANCA (reference range: <3.5 IU/mL) and PR3-ANCA autoantibodies (reference range: <2 IU/mL) were measured by immunoassay (ImmunoCAP 250, Thermo Fisher Scientific, Waltham, MA, USA). Plasma concentrations of human complement components C3c (9D9621, Abbott, Chicago, IL, USA) and C4 (9D9721, Abbott, Chicago, IL, USA) were determined by turbidimetric measurements on the ARCHITECT-C module. Reference range plasma concentrations for circulating C3c are defined as being between 0.82 to 1.93 g/L, and C4 between 0.15 to 0.57 g/L.

### 4.4. Renal Histopathology

Independent evaluation of all renal biopsies was performed by two renal pathologists, who were blinded to data assessment and data analysis. Within each renal biopsy specimen, each glomerulus was separately evaluated for present crescents, global sclerosis, and necrosis. The percentage of glomeruli with any of these pathologies was calculated as a fraction of the total number of glomeruli in each renal biopsy. Renal biopsies were scored in accordance with the current version of the Banff score for allograft pathology, as recently described [47,48]. Kidney sections (formalin-fixed, paraffin-embedded) were deparaffinized in xylene and then rehydrated in ethanol containing distilled water. Utilizing antibodies against C3c (1:10,000, A0062, Agilent Dako, Santa Clara, CA, USA) and C4d (1:50, 503-17344, Zytomed, Berlin, Germany), tissue sections were stained. According to the manufacturer’s protocol, labeling was conducted using the Novolink^TM^ Polymer Detection System (Leica Biosystems, Wetzlar, Germany). Nuclear counterstaining was performed using Mayer’s Hematoxylin Solution (Sigma, St. Louis, MO, USA). C3c and C4d deposits in the glomerular tuft, interlobular arteries, peritubular capillaries, and venules were evaluated for presence, as recently described [12,41].

### 4.5. Statistical Analysis

Utilizing the Shapiro–Wilk test, continuous variables were tested for Gaussian distribution. Statistical comparisons were not formally powered or prespecified. Non-normally distributed variables are presented as median (IQR), and normally distributed variables are itemized as mean ± standard deviation (SD). Subgroup comparison between the PR3-ANCA and MPO-ANCA subgroup was performed by means of unpaired *t* test for normally distributed variables, a Mann–Whitney test for non-normally distributed variables, and a Chi-square test for categorical variables. Pearson’s correlation was performed for assessment of correlations between continuous variables, and Spearman’s correlation was performed for correlations between categorical and continuous variables. A Pearson’s/Spearman’s r of ±0.4 (approximated) in the correlation matrix was defined as relevant, and independent statistical evaluation of these parameters was performed by means of univariable linear regression analysis, with serum UA levels as the outcome variable. To analyze the greatest effect of independent variables on the dependent variable serum UA levels, the β coefficient, also called the standardized regression coefficient, was used. Finally, all variables with a β coefficient of greater than ±0.2 in the univariable regression analyses were included in the stepwise multivariable linear regression analyses. Covariates were retained as significant differences in the linear regression model to avoid model overfitting. Heatmaps reflect the mean values of Spearman’s/Pearson’s r, significant *p* values of Pearson’s/Spearman’s correlation are marked with an asterisk, and circle size represents significance level in the stepwise multivariable linear regression. In addition, simple linear regression was performed. Probability values (*p* values) below 0.05 were considered statistically significant. Data analyses were performed with GraphPad Prism (version 9.4.0 for MacOS, GraphPad Software, San Diego, CA, USA) and IBM SPSS Statistics (version 29 for MacOS, IBM Corporation, Armonk, NY, USA).

## 5. Conclusions

We herein show that UA metabolism might be involved in activation of the complement system, and therefore broaden our current understanding of contributory metabolic factors that influence the initial disease course, especially in severe ANCA-associated renal vasculitides.

## Figures and Tables

**Table 1:** Baseline characteristics of the total cohort, CI, PR3- and MPO-ANCA subgroups, and group comparisons between the MPO-ANCA and PR3-ANCA subgroups.

	Units	Total *n* = 34	CI *n* = 19	MPO-ANCA *n* = 21	PR3-ANCA *n* = 13	*p* Value
Clinical data						
BMI	kg/m^2^	26 ± 5	25 ± 4	28 ± 4	24 ± 4	0.0417
Female sex	*n* (%)	14 (41.2)	7 (36.8)	7 (33.3)	7 (53.8)	0.2376
Age	years	69 (57–75)	72 (61–76)	66 (52–71)	74 (64–76)	0.1002
Sys. BP at kidney biopsy	mmHg	129 ± 12	129 ± 9	130 ± 12	127 ± 14	0.5754
Dia. BP at kidney biopsy	mmHg	74 ± 12	73 ± 12	76 ± 9	71 ± 16	0.4468
BVAS	Score	18 ± 4	18 ± 4	18 ± 4	18 ± 2	0.3964
SAPS II	Score	26 (21–32)	32 (25–36)	24 (21–33)	28 (22–33)	0.3041
Hyperuricemia	*n* (%)	9 (26.5)	7 (36.8)	6 (28.6)	3 (23.1)	0.6357
Decompensated DM	*n* (%)	2 (5.9)	1 (5.3)	2 (9.5)	0 (0)	0.2514
Arterial hypertension	*n* (%)	29 (85.3)	15 (78.9)	20 (95.2)	9 (69.2)	0.0375
Current alcohol abuse	*n* (%)	3 (8.8)	1 (5.3)	3 (14.3)	0 (0)	0.1535
Serum parameters						
Albumin	g/dL	2.6 ± 0.6	2.4 ± 0.7	2.8 ± 0.7	2.5 ± 0.6	0.6458
ALT	IU/L	18 (9–44)	24 (10–47)	18 (11–27)	22 (6–55)	0.9396
AP	IU/L	84 (68–108)	84 (68–124)	87 (73–106)	73 (58–138)	0.3290
AST	IU/L	26 (19–32)	27 (19–35)	27 (20–31)	20 (16–34)	0.1726
BUN	mg/dL	53 ± 28	65 ± 25	54 ± 26	51 ± 32	0.8396
C3	g/L	1.2 ± 0.3	1.1 ± 0.3	1.2 ± 0.3	1.2 ± 0.3	0.7841
C4	g/L	0.2 ± 0.1	0.2 ± 0.1	0.3 ± 0.1	0.2 ± 0.1	0.7469
C-reactive protein	mg/dL	61 (28–94)	73 (47–104)	64 (32–114)	33 (15–75)	0.1288
Serum creatinine	mg/dL	2.9 (1.4–4.9)	4.2 (2.0–6.3)	3.9 (1.4–5.0)	2.0 (0.9–4.5)	0.8064
eGFR	mL/min/1.73 m^2^	17 (9–49)	12 (9–25)	16 (9–43)	25 (12–85)	0.9609
Ferritin	µg/L	375 (268–1255)	407 (336–1869)	362 (172–972)	407 (306–1915)	0.2844
Haptoglobin	g/L	2.2 ± 1.2	2.1 ± 1.3	2.5 ± 0.2	2.0 ± 1.6	0.6690
Hemoglobin	g/dL	9.5 (8.1–10.6)	8.3 (7.7–9.4)	9.9 (8.6–11.1)	9.3 (7.8–10.2)	0.2898
Iron	µmol/L	10.9 ± 6.5	11.2 ± 6.7	12.9 ± 7.1	7.6 ± 3.9	0.1468
Lactate dehydrogenase	IU/L	264 (216–291)	263 (183–314)	230 (186–293)	268 (250–299)	0.2991
MPO-ANCA	IU/mL	27 (0.2–90)	57 (0.2–123)	71 (28–129)	0.2 (0.2–0.2)	0.0055
Platelets	10^3^/µL	279 (177–383)	213 (150–321)	253 (169–358)	320 (191–464)	0.5088
PR3-ANCA	IU/mL	0.5 (0.2–32)	0.4 (0.2–32)	0.2 (0.2–0.4)	35 (22–86)	0.0008
Procalcitonin	mg/dL	0.2 (0.1–0.5)	0.3 (0.2–0.6)	0.2 (0.1–0.6)	0.2 (0.1–0.5)	0.8643
Uric acid	µg/L	6.8 ± 1.7	6.8 ± 1.7	6.8 ± 1.7	6.4 ± 1.4	0.9999
WBC	k/µL	12 ± 5	12 ± 5	11 ± 4	13 ± 6	0.9838
Treatment						
XO inhibitors	*n* (%)	5 (14.7)	5 (26.3)	4 (19.0)	1 (7.7)	0.3636
Diuretics	*n* (%)	18 (52.9)	11 (57.9)	14 (66.7)	4 (30.8)	0.0416
Antihypertensives	*n* (%)	2 (5.9)	2 (10.5)	3 (4.3)	1 (7.7)	0.5620
Statins	*n* (%)	6 (17.6)	4 (21.1)	3 (14.3)	3 (23.1)	0.5135
Cyclophosphamide	*n* (%)	19 (55.9)	11 (57.9)	12 (57.1)	7 (53.8)	0.8508
Steroid pulse	*n* (%)	25 (73.5)	17 (89.5)	16 (76.2)	9 (69.2)	0.6549
Rituximab	*n* (%)	18 (52.9)	7 (36.4)	11 (52.4)	7 (53.8)	0.9337
Plasma exchange	*n* (%)	17 (50.0)	12 (63.2)	10 (47.6)	7 (53.8)	0.7242
KRT	*n* (%)	13 (38.2)	12 (63.2)	8 (38.1)	5 (38.5)	0.9830
Glomerular lesions						
Normal	% of total	0.4 ± 0.3	0.4 ± 0.3	0.4 ± 0.3	0.6 ± 0.2	0.0094
Necrosis	% of total (IQR)	0.3 (0.0–0.5)	0.2 (0.0–0.7)	0.3 (0.0–0.6)	0.2 (0.1–0.5)	0.8113
Crescents	% of total	0.4 ± 0.3	0.4 ± 0.3	0.5 ± 0.3	0.3 ± 0.3	0.2187
Sclerosis	% of total (IQR)	0.04 (0.0–0.3)	0.05 (0.0–0.3)	0.04 (0.0–0.4)	0.04 (0.0–0.2)	0.4053
Banff lesions						
*ah* (0/1/2/3/x)	Score	22/5/2/2/3	15/2/0/1/1	11/2/1/2/5	10/1/1/0/1	0.5009
*ci* (0/1/2/3/x)	Score	5/16/11/2/0	2/9/5/2/1	1/7/7/2/4	4/6/3/0/0	0.0901
*ct* (0/1/2/3/x)	Score	0/21/9/1/3	2/12/3/1/1	0/10/6/1/4	2/8/2/0/1	0.2450
*g* (0/1/2/3/x)	Score	6/2/20/4/2	5/1/10/2/1	6/0/9/2/4	0/2/8/2/1	0.0766
*i* (0/1/2/3/x)	Score	25/7/0/0/2	12/6/0/0/1	12/5/0/0/4	10/2/0/0/1	0.4804
*i-IFTA* (0/1/2/3/x)	Score	0/7/9/16/2	0/1/8/8/2	0/3/6/8/4	0/1/2/8/2	0.5958
*ptc* (0/1/2/3/x)	Score	28/4/0/0/2	15/3/0/0/1	16/1/0/0/4	10/2/0/0/1	0.4207
*t* (0/1/2/3/x)	Score	9/19/3/1/2	4/10/3/1/1	4/9/3/1/4	4/8/0/0/1	0.3128
*ti* (0/1/2/3/x)	Score	8/18/6/0/2	5/9/4/0/1	2/10/5/0/4	5/6/1/0/1	0.1608
*t-IFTA* (0/1/2/3/x)	Score	9/23/0/0/2	6/12/0/0/1	4/13/0/0/4	5/7/0/0/1	0.3799
*v* (0/1/2/3/x)	Score	18/4/0/5/7	9/4/0/5/1	9/2/0/3/7	9/0/0/2/2	0.3509
C3c deposits						
Glomerular tuft	*n* (%)	23 (67.6)	15 (78.9)	11 (52.4)	10 (76.9)	
Interstitial arteries	*n* (%)	1 (2.9)	0 (0)	1 (4.8)	0 (0)	
Peritubular capilliaries	*n* (%)	22 (64.7)	14 (73.7)	11 (52.4)	9 (69.2)	
Venules	*n* (%)	0 (0.0)	0 (0)	0 (0)	0 (0)	
Tubular	*n* (%)	11 (32.4)	5 (26.3)	7 (33.3)	3 (23.1)	0.6467
C4d deposits						
Glomerular tuft	*n* (%)	17 (50.0)	12 (63.2)	9 (42.9)	6 (46.2)	
Interstitial arteries	*n* (%)	11 (32.4)	8 (42.1)	6 (28.6)	4 (30.8)	
Peritubular capilliaries	*n* (%)	15 (44.1)	10 (52.6)	8 (38.1)	5 (38.5)	
Venules	*n* (%)	9 (26.5)	7 (36.4)	6 (28.6)	2 (15.4)	
Tubular	*n* (%)	23 (67.6)	14 (73.7)	13 (61.9)	8 (61.5)	0.9621

Continuous variables were tested for normal distribution utilizing the Shapiro–Wilk test. Normally distributed continuous variables are presented as mean ± SD, and non-normally distributed continuous variables are itemized as median (IQR). Subgroup comparisons were performed between MPO-ANCA and PR3-ANCA by means of an unpaired *t* test or Mann–Whitney test. Between-group comparisons for categorical variables were performed with Chi-square test. Abbreviations: *ah*, arteriolar hyalinosis; ALT, alanine aminotransferase; ANCA, anti-neutrophil cytoplasmic antibody; AP, alkaline phosphatase; AST, aspartate aminotransferase; BMI, body mass index; BP, blood pressure; BUN, blood urea nitrogen; BVAS, Birmingham Vasculitis Activity Score; C3, complement factor 3; C4, complement factor 4; CI, critically ill; *ci*, interstitial fibrosis; *ct*, tubular atrophy; dia., diastolic; DM, diabetes mellitus; eGFR, estimated glomerular filtration rate; *g*, glomerulitis; *i*, interstitial inflammation; *IFTA*, interstitial fibrosis/tubular atrophy; *i-IFTA*, inflammation in areas of IFTA; IQR, interquartile range; KRT, kidney replacement therapy; MPO, myeloperoxidase; n, number; PR3, proteinase 3; *ptc*, peritubular capillaritis; SAPS II, Simplified Acute Physiology Score; sys., systolic; *t*, tubulitis; *ti*, total inflammation; *t-IFTA*, tubulitis in areas of IFTA; *v*, intimal arteritis; WBC, white blood cells; x, excluded/not available; XO inhibitors, xanthine oxidase inhibitors.

**Table 2:** Univariable and stepwise multivariable linear regression analyses of clinical and laboratory parameters, with serum UA levels as a dependent variable. Pearson’s/Spearman’s r and *p* values are also itemized.

Variable	Pearson’s/Spearman’s		Univariable		Stepwise Multivariable	
	r	*p* Value	β	*p* Value	β	*p* Value
Total						
C3	−0.595	0.002	−0.595	0.002	−0.462	0.005
BMI	0.426	0.030	0.426	0.030	−0.184	0.241
Albumin	0.426	0.078	0.426	0.078	0.209	0.181
CI						
C3	−0.703	0.007	−0.577	0.010	−0.700	<0.001
Diuretics	0.434	0.063	0.500	0.029	0.240	0.224
XO inhibitors	0.371	0.118	0.444	0.057	0.284	0.115
BMI	0.734	0.010	0.453	0.051	0.429	0.009
Albumin	0.500	0.098	0.340	0.154	0.194	0.289
MPO-ANCA						
Ferritin	0.907	0.001	0.502	0.020	0.598	0.004
Platelets	−0.478	0.033	−0.472	0.031	−0.256	0.170
XO inhibitors	0.522	0.015	0.443	0.044	0.360	0.076
C3	−0.597	0.015	−0.462	0.035	−0.244	0.203
ALT	−0.402	0.123	−0.308	0.174	−0.359	0.049
BMI	0.363	0.167	0.301	0.185	0.125	0.561
PR3-ANCA						
Albumin	0.555	0.154	0.461	0.113	0.267	0.509
C3	−0.596	0.091	−0.506	0.078	−0.433	0.302
LDH	0.480	0.191	0.447	0.126	0.112	0.775
PEX	−0.454	0.138	−0.376	0.205	0.543	0.430
KRT	−0.465	0.127	−0.398	0.178	−0.292	0.590
BMI	0.553	0.097	0.355	0.234	0.451	0.251
CYC	−0.536	0.073	−0.439	0.133	−0.413	0.364

Independent statistical evaluation of variables offering a Pearson’s r/Spearman’s r of more than ±0.4 (approximated) in the correlation matrix were tested by means of univariable linear regression. Variables with a β coefficient of greater than ±0.2 in the univariable regression were included in the stepwise multivariable linear regression analysis to identify the strongest association with serum UA levels in the total cohort and the three subgroups. Abbreviations: ALT, alanine aminotransferase; ANCA, anti-neutrophil cytoplasmic antibody; BMI, body mass index; C3, complement factor 3; CI, critically ill; CYC, clyclophosphamide; KRT, kidney replacement therapy; LDH, lactate dehydrogenase; MPO, myeloperoxidase; PEX, plasma exchange; PR3, proteinase 3; UA, uric acid; XO inhibitors, xanthine oxidase inhibitors; β, beta coefficient.

**Table 3:** Univariable and stepwise multivariable linear regression analyses of histopathological items, with serum UA levels as a dependent variable. Pearson’s/Spearman’s r and *p* values are also itemized.

Variable	Pearson’s/Spearman’s		Univariable		Stepwise Multivariable	
	r	*p* Value	β	*p* Value	β	*p* Value
Total						
*t-IFTA*	0.524	0.002	0.508	0.003	0.494	0.008
C4d venules	0.380	0.067	0.426	0.038	0.500	0.007
Sclerosis	0.315	0.069	−0.066	0.713		
CI						
*t-IFTA*	0.443	0.066	0.489	0.040	0.488	0.034
C4d venules	0.356	0.199	0.403	0.136	0.513	0.016
C4d ptc	0.541	0.042	0.480	0.070	0.180	0.472
*i*	−0.443	0.065	−0.370	0.131	−0.470	0.021
Sclerosis	0.399	0.091	0.399	0.091	0.416	0.046
MPO-ANCA						
*t-IFTA*	0.590	0.008	0.533	0.019	0.488	0.029
C3c tubules	0.360	0.213	0.187	0.430	0.093	0.672
*t*	0.306	0.202	0.281	0.244	0.046	0.848
PR3-ANCA						
*ci*	0.484	0.107	0.626	0.022	0.626	0.022
*ti*	0.307	0.325	0.402	0.195	−0.203	0.597
*t-IFTA*	0.358	0.283	0.504	0.095	0.032	0.934
C4d venules	0.518	0.222	0.481	0.190	0.317	0.189
Sclerosis	0.429	0.144	0.429	0.144	0.462	0.043
*ptc*	−0.324	0.364	−0.164	0.611		
*i*	−0.324	0.364	−0.164	0.611		

Independent statistical evaluation of variables offering a Pearson’s/Spearman’s r more than ±0.4 (approximated) in the correlation matrix were tested by means of univariable linear regression. Variables in the univariable regression with a β coefficient of greater than ±0.2 were included in the stepwise multivariable linear regression analysis to identify the greatest association with serum UA levels in the total cohort and three subgroups. Abbreviations: ANCA, anti-neutrophil cytoplasmic antibody; C3c, complement factor 3 convertase; C4d, complement factor 4d; C4d ptc, complement factor 4d in peritubular capillaries; CI, critically ill; *ci*, interstitial fibrosis; *i*, interstitial inflammation; MPO, myeloperoxidase; PR3, proteinase 3; *ptc*, peritubular capillaritis; *t*, tubulitis; *ti*, total inflammation; *t*-*IFTA*, tubulitis in areas of IFTA; UA, uric acid; β, beta coefficient.

## Data Availability

The original contributions presented in the study are included in the article; further data and material are available from the corresponding author upon reasonable request.
